# Comparative Evaluation on the Bioaccessibility of Citrus Fruit Carotenoids In Vitro Based on Different Intake Patterns

**DOI:** 10.3390/foods11101457

**Published:** 2022-05-17

**Authors:** Yang Xu, Tan Hu, Haijuan Hu, Sihui Xiong, Kaixin Shi, Nawei Zhang, Qier Mu, Gang Xu, Peipei Zhang, Siyi Pan

**Affiliations:** 1 College of Food Science and Technology, Huazhong Agricultural University, Wuhan 430070, China; 2018309110049@webmail.hzau.edu.cn (Y.X.); hutan@webmail.hzau.edu.cn (T.H.); huhaijuan@webmail.hzau.edu.cn (H.H.); sihuixiong@webmail.hzau.edu.cn (S.X.); skx@webmail.hzau.edu.cn (K.S.); zhangnawei@webmail.hzau.edu.cn (N.Z.); qiermu@webmail.hzau.edu.cn (Q.M.); xgang@webmail.hzau.edu.cn (G.X.); zhangpeipei1217@webmail.hzau.edu.cn (P.Z.); 2 Key Laboratory of Environment Correlative Dietology, Ministry of Education, Huazhong Agricultural University, Wuhan 430070, China; 3 Hubei Key Laboratory of Fruit and Vegetable Processing and Quality Control, Huazhong Agricultural University, Wuhan 430070, China

**Keywords:** bioaccessibility, carotenoids, intake pattern, citrus, not-from-concentrate

## Abstract

The intake pattern has a great impact on the bioaccessibility of carotenoids from citrus fruit. Here, we compared the bioaccessibility of carotenoids from fresh citrus fruit (FC), fresh citrus juice (FCJ), and not-from-concentrate citrus juice (NCJ) and analyzed the influencing factors. The results demonstrated that particle size, viscosity, and some active components of the samples during digestion are potential factors affecting the bioaccessibility of carotenoids. The total carotenoid bioaccessibility of NCJ (31.45 ± 2.58%) was significantly higher than that of FC (8.11 ± 0.43%) and FCJ (12.43 ± 0.49%). This work demonstrates that NCJ is an appropriate intake pattern to improve the bioaccessibility of carotenoids from citrus fruit. The findings also suggest that adjustment of food intake patterns is an effective way to improve the digestion and absorption of nutrients.

## 1. Introduction

Carotenoids, which are natural pigments widely present in plants, have important functional properties such as provitamin activity and antioxidant activity [1]. They have also been demonstrated to improve the immune system and be able to prevent the occurrence of chronic degenerative diseases [2]. For example, β-carotene can be metabolized to vitamin A by enzymes in the liver and intestinal walls in humans and animals [3]. In addition, two dietary carotenoids, lutein and zeaxanthin, play vital roles in the human retina, protecting the retina from photochemical damage with certain antioxidant properties, such as preventing UV-induced peroxidation and reducing lipofuscin formation and related oxidative stress-induced damage [4]. However, carotenoids cannot be synthesized by humans, and can only be absorbed from daily food such as fruits and vegetables.

Citrus fruit are economically important and often consumed as minimally processed or further processed products (such as juice and puree). To date, citrus fruit have been identified to contain about 115 types of carotenoids [5], such as α-carotene, β-citraurin, β-apo-8′-carotene aldehyde, β-carotene, β-cryptoxanthin, auroxanthin, luteoxinthin, lutein, mutatoxinthin, lycopene, phytoene, phytofluene, zeaxanthin, neoxanthin, violaxanthin, and ζ-carotene [6]. Although citrus fruit and its processed products can provide essential carotenoids through daily diet, their effects are largely limited by their low bioaccessibility. It has been demonstrated that the factors affecting the bioaccessibility of carotenoids include carotenoid type, molecular interaction between different types of carotenoids, the content of carotenoids, food matrix containing carotenoids, effectors of absorption and nutrients, and some genetic factors [7]. For example, a high concentration of pectin can inhibit the micellization of carotenoids, while a low pectin concentration promotes carotenoid micellization; β-lactoglobulin can promote carotenoid transport but inhibit lipase activity [8]. Great research efforts have been made to improve the bioaccessibility of carotenoids. It has been demonstrated that processing can modify the chemical structures and properties of carotenoids, which can contribute to the improvement of carotenoid bioaccessibility.

It has been reported that citrus processing, such as heating, ultrasound, and homogenization treatments, can affect the species, content, structure, and bioaccessibility of carotenoids. Sentandreu et al. (2020) reported that heat treatment could reduce total carotenoids in Ortanique mandarin juice relative to fresh juice. However, fresh and pasteurized juice exhibited similar bioaccessibility to total carotenoids [9]. Later, Wellala et al. (2022) illustrated that high-pressure homogenization (HPH) could enhance the bioaccessibility of total carotenoids in mixed juice [10]. Zhang et al. (2019) demonstrated that ultrasound treatment at 800 W could increase the bioaccessibility of total lycopene by 1.76 times [11]. In general, most previous studies have focused on the effect of heat or non-heat treatment on the bioaccessibility of carotenoids in juice. As a matter of fact, adjustment of intake patterns may be another effective way to promote the release of carotenoids and their bioaccessibility. The intake patterns of citrus fruit mainly include direct eating and consumption of citrus fruit juice. However, there have been few studies comparing the effects of different intake patterns of citrus fruit on the bioaccessibility of carotenoids. Generally, citrus juice on the market can be divided into concentrated citrus juice and not-from-concentrate citrus juice (NCJ). NCJ is produced through homogenization and sterilization (without concentration and recycling) of fresh citrus juice (FCJ). NCJ is not only closer to fresh fruit in taste and flavor but also retains more nutrients such as polyphenols and carotenoids [12,13]. Therefore, a comparison of the carotenoid bioaccessibility among fresh citrus fruit (FC), FCJ, and NCJ can help to guide the customers in daily diet for the supplement of carotenoids.

## 2. Materials and Methods

### 2.1. Chemicals and Reagents

Methyl tert-butyl ether (MTBE) and Methanol (MeOH) of HPLC grade were obtained from Thermo Fisher Scientific (Leicestershire, UK). Ptyalize, trypsin (porcine pancreas), pepsin (porcine gastric mucosa), and bile salts to be used in vitro were purchased from Sigma-Aldrich (St. Louis, MO, USA). The β-carotene standards were provided by Yuanye Bio-Technology Co., Ltd. (Shanghai, China). Violaxanthin and antheraxanthin were obtained from Sigma (St. Louis, MO, USA). All other chemicals of analytical grade were provided by Sinopharm chemical reagent Co., Ltd. (Shanghai, China).

### 2.2. Sample Preparation

Commercially mature navel citrus “Jiuyuehong” fruit were purchased from Zigui County, Yichang City, Hubei Province, China in 2020. The fruit were rinsed, peeled and roughly broken with a wall-breaking machine (SP528, Supor, Hangzhou, China) to simulate chewing, which was taken as fresh citrus group (FC). The fruit were then extruded by a household juicer (HU-910IV-M, HUROM, Shanghai, China) and filtered through a steel sieve (1 mm), which was taken as fresh citrus juice group (FCJ). The fresh citrus juice was homogenized twice with a homogenizer (ATS Engineering Inc., AH100B, Brampton, ON, Canada) at 80 MPa, and immediately pasteurized (80 °C, 15 min) after vacuum encapsulation (Ding Gong, DG500, Wuhan, China) to be used as not-from-concentrate citrus juice group (NCJ). All the obtained samples were stored at −80 °C for later analysis.

### 2.3. Determination of Vitamin C (VC) Content

In an acetic acid solution, ascorbic acid could react with fast blue B salt to produce yellow derivative. By measuring the absorbance value at 420 nm and comparison with the calibration curve, the ascorbic acid content could be measured. In detail, after fully mixing 0.2 g ascorbic acid and 20 mL acetic acid solution (2 mol/L), the VC reserve solution (2.0 g/L) was obtained in a 100-mL brown volumetric flask with distilled water. After fully mixing 5.0 mL VC reserve solution and 5.0 mL acetic acid solution (2 mol/L), the VC calibration solution (0.1 g/L) was obtained by constant volume of distilled water in a 100 mL brown volumetric flask. After the sample (20 g) was mixed with acetic acid solution (2 mol), it was kept in a 100-mL-brown volumetric flask as the liquid to be tested. VC standard solution (0.00, 0.10, 0.20, 0.40, 0.60, 0.80, 1.0, 1.5, and 2.0 mL, respectively), and 1 mg of the sample solution were taken to be tested, and 0.3 mL EDTA solution, 0.5 mL acetic acid solution (0.5 mol) and 1.25 mL solid blue salt B solution (2 g/L) were added successively, and mixed fully with distilled water to a constant volume of 10 mL. After 20 min of standing, the absorbance was measured at 420 nm by spectrophotometer (AOE, UV-1200, China). The calibration curve of VC concentration was plotted and the content of VC was calculated with the calibration curve (y = 0.0671x − 0.0050; R^2^ = 0.9987; limit of detection, 0.0–20.0 μg/mL).

### 2.4. Determination of Total Phenolics

The Folin–Ciocalteu method was used as previously described with minor modifications [14]. Gallic acid was prepared by adding deionized water into a calibration solution of 0, 25, 50, 100, 150, 200 μg/mL gallic acid. The samples were configured with deionized water to form a 0.2 g/mL solution for the samples to be tested. About 0.2 mL standard/sample solution was mixed with 1 mL Folin–Ciocalteu reagent and 0.8 mL deionized water, and the mixture was allowed to stand at room temperature for 5 min, followed by the addition of 1 mL Na_2_CO_3_ solution (75%). The absorbance was detected at 760 nm with a spectrophotometer (AOE, UV-1200, Shanghai, China) after reacting at room temperature for 1 h. Total phenolics were quantified with calibration curve generated from gallic acid standard solutions (y = 0.0052x + 0.3620; R^2^ = 0.9993, limit of detection, 0.0–13.3 μg/mL), and the content was expressed as gallic acid equivalent mg gallic acid equivalents (GAE)/100 g of samples.

### 2.5. In Vitro Digestion

Simulated saliva fluid (SSF), simulated gastric fluid (SGF), and simulated intestinal fluid (SIF) were prepared as previously described [15]. All solutions were heated to 37 °C before each digestion phase and then held at 37 °C throughout the experimental period.

#### 2.5.1. Mouth Phase Digestion

About 5 g sample was mixed with 4 mL of SSF and 25 μL of CaCl_2_ (0.3 M), followed by the addition of salivary amylase to a final concentration of 75 U/mL. The total volume was adjusted to 10 mL by the addition of deionized water, followed by incubation under darkness at 37 °C for 2 min.

#### 2.5.2. Stomach Phase Digestion

The mouth digesta was then mixed with 8 mL SGF and 5 μL CaCl_2_ (0.3 M). The pH was adjusted to 3.0 by adding 1 M of HCl. Subsequently, porcine pepsin solution was added to achieve an activity of 2000 U/mL in the digestion mixture. Then, the total volume was made into 20 mL with deionized water, followed by incubation under darkness at 37 °C for 2 h.

#### 2.5.3. Small Intestine Phase Digestion

The stomach digesta was mixed with 16 mL of SIF and 40 μL of CaCl_2_ (0.3 M). The pH was adjusted to 7.0 by the addition of NaOH solution (1 M). Then, the pancreatin solution was added with SIF to make a final concentration of 10 mM and 100 U/mL for pancreatin and bile salts, respectively. The total volume was subsequently adjusted to 40 mL by the addition of deionized water, followed by incubation under darkness at 37 °C for 2 h. The small intestinal digestion was stopped by placing the samples in liquid nitrogen.

### 2.6. Extraction of Carotenoids

The extraction of carotenoids was performed with previously reported methods with minor modifications [16,17]. Briefly, samples were fully mixed with extraction solution (N-hexane: ethanol: acetone = 2:1:1, *v*/*v*, 0.1% BHT) in a ratio of 1:2 (*w*/*v*). Subsequently, stirring of the mixture was conducted at 700 rpm for 30 min and centrifugation was performed at 7000 rpm for 3 min for the collection of supernatant. The extraction was repeated three times until the residue was colorless. Finally, the supernatant was incorporated for saponification.

Saponification was carried out with the improved methods from previous studies [18]. The extract was washed with distilled water to remove ethanol, and the remaining extract was mixed with 20% (*w*/*v*) ethanol/potassium hydroxide solution of the same volume and placed under darkness at room temperature for 1 h. The saponified extract was washed with distilled water three times and then washed with 10% NaCl. The saponified samples were evaporated to dry (25 °C) by a rotary evaporator, re-dissolved in a MeOH-MTBE (1:2, *v*/*v*, 0.1% BHT) solution (2 mL), and then passed through a 0.22 μm PTFE filter to be used for subsequent HPLC analysis.

### 2.7. HPLC Analysis

HPLC analysis was performed by referring to a previously reported method [2]. The extract was analyzed with a 2695 HPLC system (Waters Corp., Milford, MA, USA) equipped with a C-30 reversed-phase column (250 × 4.6 mm, 5 μm) and a C-30 guard column (20 × 4.0 mm, 5 μm) (YMC, Inc., Wilmington, NC, USA). Gradient elution was carried out by using methanol/MTBE/distilled water (81:15:4, *v*/*v*/*v*) and MTBE/methanol (90:10, *v*/*v*) as the mobile phase. The linear gradient program was set as follows: 0 min: 100% A; 25 min: 75% A; 80 min: 15% per type; 82 min: 100% A. The flow rate was set at 1 mL/min, and the UV–VIS spectra were collected at 450 nm. The column temperature was maintained at 25 °C and a 20 μL sample was injected for analysis.

### 2.8. Particle size Distribution Analysis

The Malvern Zetasizer Nano ZS analyzer (ZEN 3600, Malvern Instrument Co., Ltd., Worcestershire, UK) was used to measure the particle size distribution in intestinal and micellar media by dynamic light scattering. The index of refraction for digestive juice used to calculate particle size was 1.47 and that for water was 1.33.

### 2.9. Determination of Viscosity

A rotational rheometer (Discovery HR-2, TA Instruments, New Castle, DE, USA) was employed to measure the viscosity of gastric and intestinal media, and a parallel plate geometry (40 mm diameter) with a gap size of 1 mm was used. The shear rates were 0.1 to 1000 s^−1^. The viscosity curve (apparent viscosity in relation to the shear rate) was recorded.

### 2.10. Calculation of Carotenoid Retention Ratio (CRR)

The calculation formula of carotenoid retention ratio (CRR) in the small intestine has been described previously [19]:

CRR (%) = (C_raw digesta_/C_initial_) × 100

where C_raw digesta_ and C_initial_ represent the carotenoid content in the model system after small intestine stage and in the initial model system, respectively. The CRR was expressed as percentage.

### 2.11. Calculation of Carotenoid Bioaccessibility (CBA)

After the small intestine stage, the digesta was subjected to centrifugation at 10,000 rpm and 4 °C for 1 h (Centrifuge 5804R, Eppendorf, Hamburg, Germany), resulting in bottom precipitate and top clear supernatant. Then, the supernatant was filtered through a 0.22 μm microliter membrane to collect micelle fraction. The carotenoid bioaccessibility (CBA) was calculated as follows [20]:

CBA (%) = (C_micelle_/C_raw digesta_) × 100

where C_micelle_ and C_raw digesta_ represent the carotenoid contents in the micelle fraction and raw digesta, respectively. CBA was expressed as percentage.

### 2.12. Statistical Analysis

All experiments were performed in triplicate, and the data were expressed as the mean ± SD of three independent experiments. One-way analysis of variance (ANOVA) was employed to compare the mean values, and significant difference was taken at *p* < 0.05. All statistical analyses were carried out with IBM SPSS Statistics Version 20.0.

## 3. Results and Discussion

### 3.1. Contents of Vitamin C (VC) and Total Phenolics

**Figure 1:**
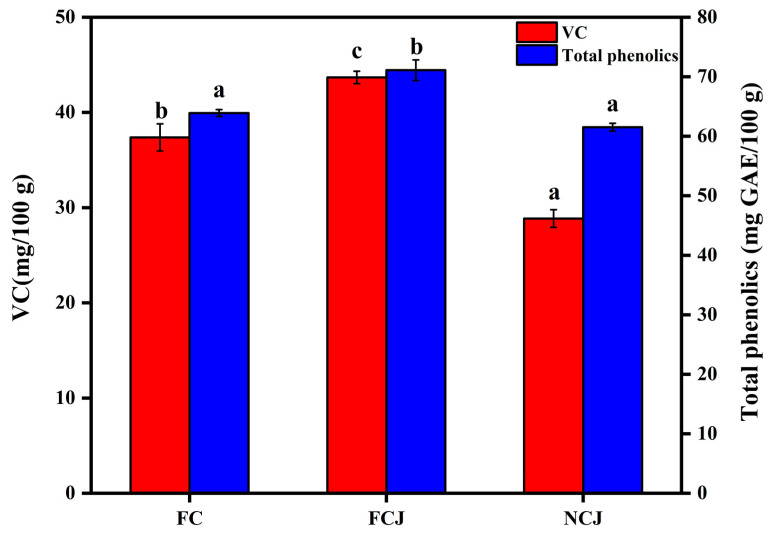
Contents of vitamin C (VC) and total phenolics in fresh citrus (FC), fresh citrus juice (FCJ), and not-from-concentrate citrus juice (NCJ). Gallic acid equivalents (GAE). Different lowercase letters indicate significant differences, *p* < 0.05.

VC and total phenolic contents are critical indicators to evaluate the quality of citrus fruit and its processed products. Figure 1 shows the VC and total phenolic contents in FC, FCJ, and NCJ. Clearly, the content of VC in FCJ (43.69 ± 0.65 mg/100 g) was higher than that in FC (37.38 ± 1.43 mg/100 g) and NCJ (28.86 ± 0.92 mg/100 g) (*p* < 0.05). VC is sensitive to environmental conditions (such as oxygen, heat, and light). Therefore, the homogenization and heat treatment for NFJ might decrease the VC content. It has been reported that heating (80 °C, 15 min) and high-pressure homogenization (HPH) can decrease the VC content [21,22]. Moreover, VC is widely distributed in plant cell matrix, and the sheer force of the juicer can break the cells so as to facilitate the release of VC. These facts can explain the higher VC content in FCJ than in FC.

A similar trend was also observed for the content of phenolics. As shown in Figure 1, the total phenolic content in FCJ (71.10 ± 1.74 mg/100 g) was higher than that in FC (63.90 ± 0.58 mg/100 g) and NCJ (61.52 ± 0.66 mg/100 g) (*p* < 0.05). Considering the complex treatment during the preparation, it is reasonable to speculate that many degradation processes occur during the production process of NCJ. Similar results were observed in citrus juice during pasteurization [21]. However, during the preparation of NCJ, HPH could inactivate the oxidases and thereby protect phenolics from autooxidation [14,23]. Therefore, it can be speculated that heat treatment has a greater impact on the content of phenolics than HPH treatment for NCJ. As for FCJ, the soft shearing process tends to break the pulp cells and release more phenolics, contributing to the highest content of total phenolics. Phenolics and carotenoids are two main secondary metabolites in citrus fruit. It has been demonstrated that phenolics can inhibit the oxidation of carotenoids in the citrus juice matrix [24]. Therefore, the phenolic content is closely related to the carotenoid content in the juice matrix.

### 3.2. Carotenoid Analysis

Table 1 summarizes the carotenoid contents (μg/g) of FC, FCJ, and NCJ. The carotenoid profile of citrus “Jiuyuehong” mainly included provitamin A carotenoids (β-cryptoxanthin and β-carotene) and other bioactive carotenoids (9-cis-violaxanthin-C12:0-C18:1, a mixture of 9-cis-violaxanthin-C12:0-C16:0 and 9-cis-violaxanthin-C14:0-C18:1, and β-cryptoxanthin-C18:0). The types of carotenoids were similar to those reported previously [6].

The total carotenoid content (TCC) of NCJ (45.16 ± 2.17 μg/g) was significantly higher than that of FC (40.75 ± 1.45 μg/g) (*p* < 0.05), indicating that the not-from-concentrate production process could promote the release of carotenoids from citrus fruit. Although the thermal treatment could lead to the oxidative degradation of carotenoids and thus decrease the carotenoid content, homogenization pressure may lead to a gradually higher carotenoid release, despite certain degradation [25]. It has been well established that carotenoids are of high sensitivity to temperature and prone to be degraded under high temperatures. Previous studies have suggested that a longer heating time and higher heating temperature will lead to more loss of carotenoids [23,26]. Similarly, HPH treatment may cause the degradation of carotenoids. On the one hand, the pulp particles and cells undergo more severe structural damage as demonstrated by the marked reduction in particle size; on the other hand, the sample is subjected to thermal treatment (80 °C, 15 min). These effects can facilitate the release of carotenoids from the food matrix and also make carotenoids more susceptible to degradation by some agents including enzymes, oxidizing agents, and acids [27]. However, the homogenization pressure may lead to a gradual increase in carotenoid release despite certain degradation [25], Moreover, multiple HPH passes tend to cause a further release of carotenoids [10]. However, NCJ showed no significant increase in carotenoids compared with FCJ (43.12 ± 0.87 μg/g) (*p* > 0.05). Therefore, it can be speculated that although the not-from-concentrate process could destroy the citrus cells and then release more carotenoids, the oxidation and degradation of carotenoids during the process would also lead to the loss of carotenoids, which may explain the insignificant changes in TCC of FCJ compared with FC and NCJ.

In addition, we also investigated the changes in single carotenoids in different samples. For provitamin A carotenoids, there was no significant change in the content of α-cryptoxanthin and β-cryptoxanthin (*p* > 0.05). The β-carotene content in FCJ (3.56 ± 0.10 μg/g) was higher than that in FC (2.99 ± 0.35 μg/g) and NCJ (3.03 ± 0.15 μg/g) (*p* < 0.05), which was possibly due to the promoted carotenoid release by juicing and the counterbalance between carotenoid degradation caused by heat treatment and carotenoid release resulting from homogenization. The changes in carotenoid content could also affect the carotenoid bioaccessibility in further digestion and adsorption.

### 3.3. Particle Size Distribution and Viscosity Analysis

**Figure 2:**
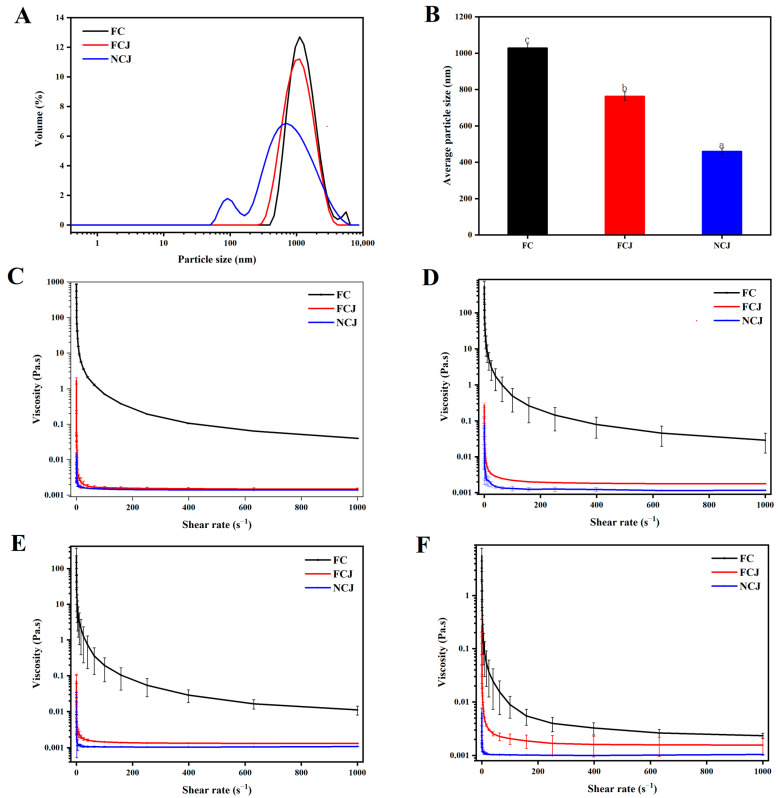
(**A**,**B**) Particle size distribution and average particle size in micellar media. (**C**–**F**) viscosity curves (apparent viscosity relative to the shear rate) of digestive fluids during digestion. (**C**) Initial phase; (**D**) Mouth phase; (**E**) Stomach phase; (**F**) Small intestine phase. Fresh citrus, fresh citrus juice, and not-from-concentrate citrus juice presented as FC, FCJ, and NCJ, respectively. Different lowercase letters represent significant differences, *p* < 0.05.

After small intestinal digestion, the carotenoids can have interactions with bile salts, phospholipids, and lipids to form a micelle. The particle size of the micelle is important for the CBA. Figure 2A,B presents the particle size distribution and average particle size of micelle in FC, FCJ, and NCJ after intestinal digestion. The particle size of FC micelle was larger than that of FCJ and NCJ micelle, which may be attributed to the flocculation of undigested fiber fragments. It could be found that NCJ had the smallest particle size among the three samples (*p* < 0.05), possibly because heat and HPH treatments contribute to the formation of carotenoids into smaller particles during micellization, which would increase the specific surface area (SSA) and thereby the absorptive rate of carotenoids [9]. Notably, the particle size distribution of NCJ micelle was multimodal, indicating that the NCJ particles were not uniform in size [28].

The apparent viscosity of three samples at different digestion phases (mouth, stomach, and small intestine digestion) was also compared in this work. The apparent viscosity decreased with increasing shear rate in all phases (Figure 2C–F), indicating a shear thinning behavior. Specifically, FC had the highest apparent viscosity at all digestion phases, indicating that a large amount of insoluble dietary fiber can significantly increase the viscosity of the digestive fluid, which is consistent with the findings in a previous study [29]. It is worth noting that the viscosity properties are dependent on the food itself and affected by the processing methods [30]. FCJ and NCJ had lower apparent viscosities, probably due to the reduction of suspended particles and shear forces received during machining. When the citrus juice is subjected to shear force, an entanglement of macromolecular polymers in the juice will be gradually reduced. With the increase in shear force, some macromolecules, (i.e., pectin) are disassembled, resulting in a decrease in viscosity. HPH can significantly decrease the viscosity of fruit juice. Previous reports have indicated that fruit juice viscosity is related to the particle size distribution, and HPH would decrease the viscosity of strawberry juice by decreasing the particle size [12,31]. However, thermal treatment was found to increase the viscosity of carrot juice due to the dissolution and flocculation of pectin and cellulose in the cell walls [32]. In general, the not-from-concentrate process reduced the viscosity of NCJ during digestion. Despite the potential and wide applicability of in vitro digestion, there are still some limitations since these models cannot fully simulate the entire process like in vivo, such as hormonal and neural control, feedback mechanisms, and peristalsis.

### 3.4. Carotenoid Retention Ratio (CRR) in the Small Intestine

**Figure 3:**
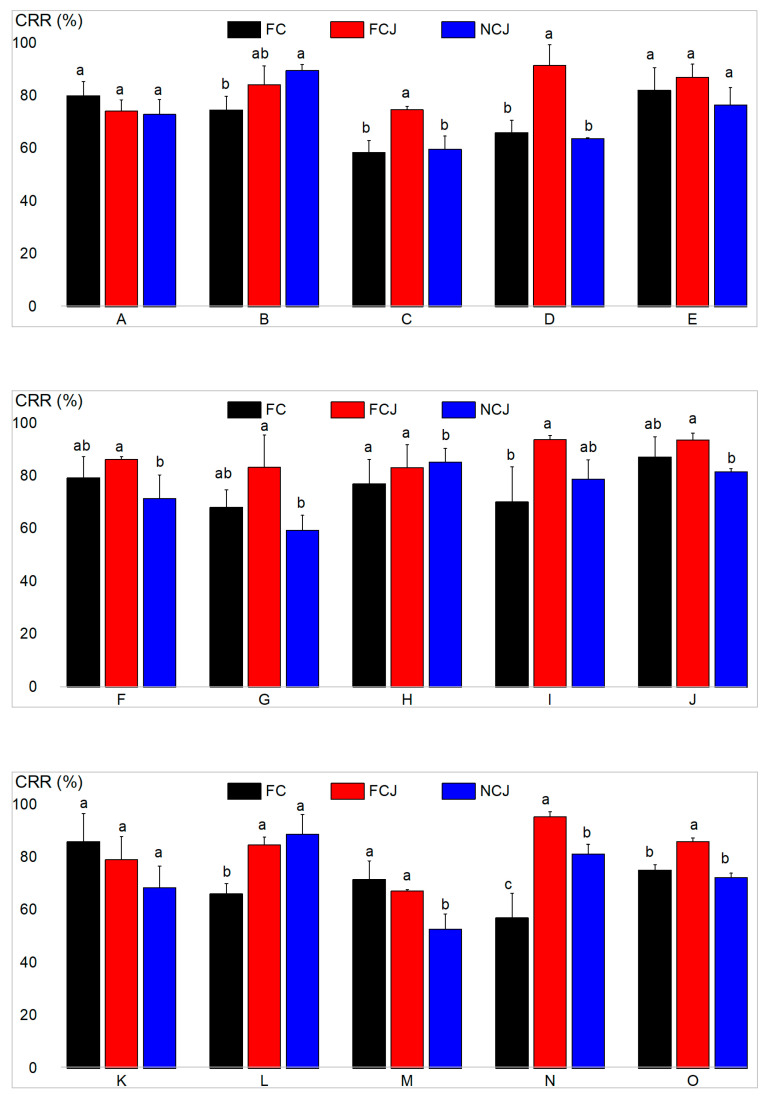
CRR of fresh citrus (FC), fresh citrus juice (FCJ) and not-from-concentrate citrus juice (NCJ). **A**: 9-cis-violaxanthin. **B**: 9-cis-antheraxanthin. **C**: 9-cis-zeaxanthin. **D**: 13- or 15-cis-β-cryptoxanthin. **E**: α-cryptoxanthin. **F**: β-cryptoxanthin. **G**: β-carotene. **H**: 9-cis-violaxanthin-C12:0- C12:0. **I**: 9-cis-violaxanthin-C12:0-C14:0. **J**: 9-cis-violaxanthin-C12:0-C18:1. **K**: 9-cis-violaxanthin-C14:0-C14:0. **L**: Mixture of 9-cis-violaxanthin-C12:0-C16:0 and 9-cis-violaxanthin-C14:0-C18:1. **M**: Mixture of violaxanthin-C14:0-C16:0 and violaxanthin-C16:0-C18:1. **N**: β-cryptoxanthin-C18:0. **O**: Total carotenoid. Different lowercase letters indicate significant differences, *p* < 0.05.

Carotenoids are known to be unstable under changes in light, acid, and temperature. Therefore, acids and other potential pro-oxidants may cause significant loss of carotenoids when passing through the digestive tract, making it important to study the digestive stability of carotenoids [33]. CRR can represent the stability of carotenoids during digestion and transport in human bodies and is also one of the important factors to be considered when studying the bioaccessibility of carotenoids [34]. Table 2 and Figure 3 present the content and CRR of carotenoids after intestinal digestion, respectively. After intestinal digestion, the total remaining carotenoid contents of FC, FCJ, and NCJ in the system were 30.67 ± 0.59, 37.12 ± 0.46, and 32.69 ± 1.11 μg/g, respectively. The CRR of total carotenoids of FCJ (86.31 ± 1.31%) was higher than that of FC (75.31 ± 2.13%) and NCJ (72.43 ± 1.66%) (*p* < 0.05). Previous studies have demonstrated that the gastric phase has the greatest contribution to the reduction of carotenoids in in vitro digestion simulation. In addition, Courraud et al. (2013) observed a 52% decrease in β-carotene in the gastric phase [33]. The high CRR of FCJ may be due to the high content of antioxidants (more phenolics and VC as mentioned in Section 3.1), which could protect carotenoids from oxidative degradation and enhance the CRR [24]. The higher CRR of α-cryptoxanthin and β-cryptoxanthin relative to that of β -carotenoids observed in all systems may indicate the occurrence of hydrolysis of carotenoid esters, which is similar to the results in a previous study [35]. Overall, the CRR of carotenoids in these three samples was related to the content of antioxidants. Previous studies have demonstrated that high levels of VC and phenolics can protect carotenoids from oxidation and degradation during digestion and transport [24].

### 3.5. Carotenoid Bioaccessibility (CBA)

**Figure 4:**
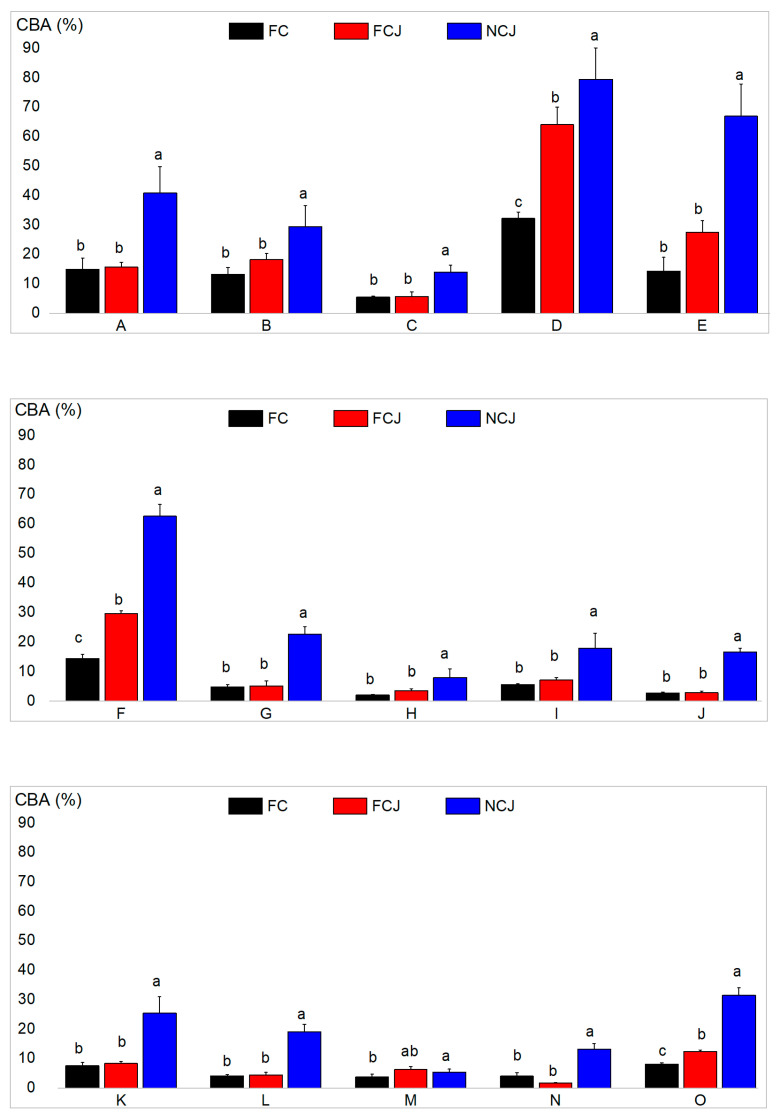
CBA of fresh citrus (FC), fresh citrus juice (FCJ) and not-from-concentrate citrus juice (NCJ). **A**: 9-cis-violaxanthin. **B**: 9-cis-antheraxanthin. **C**: 9-cis-zeaxanthin. **D**: 13- or 15-cis-β-cryptoxanthin. **E**: α-cryptoxanthin. **F**: β-cryptoxanthin. **G**: β-carotene. **H**: 9-cis-violaxanthin-C12:0- C12:0. **I**: 9-cis-violaxanthin-C12:0-C14:0. **J**: 9-cis-violaxanthin-C12:0-C18:1. **K**: 9-cis-violaxanthin-C14:0-C14:0. **L**: Mixture of 9-cis-violaxanthin-C12:0-C16:0 and 9-cis-violaxanthin-C14:0-C18:1. **M**: Mixture of violaxanthin-C14:0-C16:0 and violaxanthin-C16:0-C18:1. **N**: β-cryptoxanthin-C18:0. **O**: Total carotenoid. Different lowercase letters indicate significant differences, *p* < 0.05.

In in vitro digestion simulation, the content of carotenoids in the mixed micelles at the small intestine stage was calculated [9]. The carotenoids extracted from small intestinal supernatant were considered micellar carotenoids. Table 3 and Figure 4 show the contents of micellar carotenoids and the CBA after digestion, respectively. The total CBA in NCJ (31.45 ± 2.58%) was significantly higher than that in FC (8.11 ± 0.43%) and FCJ (12.43 ± 0.49%) (*p* < 0.05), suggesting that intake in the form of NCJ is more conducive to the formation of carotenoid micelles than that in the form of FCJ and FC. The variation in sample particle size and viscosity is one of the main reasons for the improvement of CBA. In the previous section, it has been proven that NCJ could form the smallest micelle particles, which could promote the release and micelle formation of carotenoids by decreasing the average surface area (ASA) and increasing SSA. The reduced particle size and increased SSA can make the carotenoid-containing pulp particles more accessible to digestive enzymes and promote carotenoid release during digestion [9]. The high viscosity of the digestive medium could result in slower transport of carotenoids and hinder the progress of carotenoid digestion. A previous study has also demonstrated that lower viscosity is more favorable for lipase activity and carotenoid micelle formation [36]. As for FC, the larger average particle size and higher viscosity caused by insoluble dietary fiber in the pulp can hinder the release of carotenoids and the formation of micelles. Moreover, insoluble dietary fiber can directly bind to bile acids, which is one of the factors preventing the formation of carotenoid micelles [29]. Moreover, phenolic compounds can affect the bioaccessibility of carotenoids when they are ingested together. Phenolic compounds can protect carotenoids from oxidative degradation during digestion, but at the same time will inhibit the micellization of carotenoids. In addition to carotenoids, phenolic compounds are also released from the matrix into the chyme, which will enhance the interaction of phenolics with other components such as peptides, cholesterol, and fatty acids [24]. Phenolics can affect the size and SSA of emulsion droplets [37,38], as well as reduce the binding affinity of carotenoid micelle for bile salts and the micelle solubility of cholesterol, which is an important source for the formation of mixed micelles and affects the bioaccessibility of carotenoids [38,39,40]. Compared with FC and FCJ, NCJ had a low content of phenolics, which might also be one reason for its higher carotenoid bioaccessibility.

It was noted that the increasing trend of provitamin A carotenoid bioaccessibility is similar to that of total carotenoids (NCJ had significantly higher CBA than FC and FCJ) (*p* < 0.05). Moreover, the bioaccessibility of β-carotenoids in all three samples was lower than that of α-cryptoxanthin and β-cryptoxanthin, which is similar to the finding that the bioavailability of xanthophylls in citrus is higher than that of carotenes in a recent study [41].

## 4. Conclusions

Overall, the intake pattern could affect carotenoid bioaccessibility in citrus fruit. The carotenoid content in NCJ was not negatively affected by processing. Importantly, NCJ had higher CBA than the other two samples, mainly due to the lower viscosity and the formation of micelle particles with smaller sizes. In addition, the reduction of phenolic compounds may also be an important reason for the promoted micelle formation. In conclusion, citrus fruit intake in the form of NCJ can improve carotenoid bioaccessibility. However, further research on the bioavailability of citrus carotenoids requires to be performed through Caco-2 cell uptake and animal experiments. Our results provide new insights into the effects of different intake patterns on carotenoid bioaccessibility and may be helpful in promoting the production of not-from-concentrate juice.

## Figures and Tables

**Table 1:** Carotenoid contents of fresh citrus (FC), fresh citrus juice (FCJ), and not-from-concentrate citrus juice (NCJ) (μg/g).

Carotenoids	Sample		
	FC	FCJ	NCJ
9-cis-violaxanthin	0.65 ± 0.04 b	0.77 ± 0.09 a	0.42 ± 0.01 c
9-cis-antheraxanthin	0.83 ± 0.03 a	0.65 ± 0.05 b	0.40 ± 0.05 c
9-cis-zeaxanthin	0.40 ± 0.01 a	0.36 ± 0.03 a	0.23 ± 0.03 b
13- or 15-cis-β-cryptoxanthin	0.37 ± 0.02 b	0.39 ± 0.05 b	1.23 ± 0.14 a
α-cryptoxanthin	0.63 ± 0.04 a	0.62 ± 0.11 a	0.74 ± 0.04 a
β-cryptoxanthin	12.79 ± 1.24 a	11.78 ± 0.57 a	12.94 ± 1.74 a
β-carotene	2.99 ± 0.35 b	3.56 ± 0.10 a	3.03 ± 0.15 b
9-cis-violaxanthin-C12:0-C12:0	1.17 ± 0.12 a	1.00 ± 0.04 b	0.77 ± 0.05 c
9-cis-violaxanthin-C12:0-C14:0	1.03 ± 0.11 a	1.15 ± 0.05 a	1.01 ± 0.17 a
9-cis-violaxanthin-C12:0-C18:1	6.90 ± 0.32 a	7.09 ± 0.20 a	7.24 ± 0.40 a
9-cis-violaxanthin-C14:0-C14:0	1.00 ± 0.07 b	1.28 ± 0.10 a	1.05 ± 0.11 b
Mixture of 9-cis-violaxanthin-C12:0-C16:0 and 9-cis-violaxanthin-C14:0-C18:1	5.60 ± 0.26 b	6.55 ± 0.30 a	5.60 ± 0.40 b
Mixture of Violaxanthin-C14:0-C16:0 and Violaxanthin-C16:0-C18:1	3.58 ± 0.50 b	3.53 ± 0.07 b	6.87 ± 1.08 a
β-cryptoxanthin-C18:0	2.81 ± 0.27 c	4.38 ± 0.26 a	3.61 ± 0.30 b
Total carotenoid	40.75 ± 1.45 b	43.12 ± 0.87 ab	45.16 ± 2.17 a

Data are mean ± SD. Different lowercase letters indicate significant differences, *p* < 0.05.

**Table 2:** Carotenoid retention in the small intestine of fresh citrus (FC), fresh citrus juice (FCJ), and not-from-concentrate citrus juice (NCJ) (μg/g).

Carotenoids	Sample		
	FC	FCJ	NCJ
9-cis-violaxanthin	0.52 ± 0.07 a	0.57 ± 0.05 a	0.30 ± 0.02 b
9-cis-antheraxanthin	0.62 ± 0.06 a	0.55 ± 0.03 a	0.36 ± 0.05 b
9-cis-zeaxanthin	0.23 ± 0.02 a	0.27 ± 0.02 a	0.14 ± 0.03 b
13- or 15-cis-β-cryptoxanthin	0.24 ± 0.02 c	0.36 ± 0.02 b	0.78 ± 0.09 a
α-cryptoxanthin	0.51 ± 0.03 a	0.53 ± 0.07 a	0.57 ± 0.08 a
β-cryptoxanthin	10.1 ± 0.43 ab	10.18 ± 0.62 a	9.17 ± 0.32 b
β-carotene	2.04 ± 0.21 b	2.98 ± 0.50 a	1.80 ± 0.09 b
9-cis-violaxanthin-C12:0-C12:0	0.90 ± 0.06 a	0.83 ± 0.05 a	0.66 ± 0 b
9-cis-violaxanthin-C12:0-C14:0	0.71 ± 0.07 c	1.08 ± 0.05 a	0.79 ± 0.07 b
9-cis-violaxanthin-C12:0-C18:1	6.04 ± 0.81 a	6.64 ± 0.24 a	5.92 ± 0.28 a
9-cis-violaxanthin-C14:0-C14:0	0.86 ± 0.12 ab	1.01 ± 0.05 a	0.72 ± 0.05 b
Mixture of 9-cis-violaxanthin-C12:0-C16:0 and 9-cis-violaxanthin-C14:0-C18:1	3.70 ± 0.04 c	5.55 ± 0.07 a	4.96 ± 0.13 b
Mixture of Violaxanthin-C14:0-C16:0 and Violaxanthin-C16:0-C18:1	2.59 ± 0.61 b	2.38 ± 0.03 b	3.59 ± 0.21 a
β-cryptoxanthin-C18:0	1.59 ± 0.18 c	4.18 ± 0.21 a	2.93 ± 0.11 b
Total carotenoid	30.67 ± 0.59 c	37.12 ± 0.46 a	32.69 ± 1.11 b

Data are mean ± SD. Different lowercase letters represent significant differences, *p* < 0.05.

**Table 3:** Carotenoid contents in micelles of fresh citrus (FC), fresh citrus juice (FCJ), and not-from-concentrate citrus juice (NCJ) (μg/g).

Carotenoids	Sample		
	FC	FCJ	NCJ
9-cis-violaxanthin	0.08 ± 0.01 b	0.09 ± 0.01 b	0.12 ± 0.02 a
9-cis-antheraxanthin	0.08 ± 0.01 a	0.10 ± 0.01 a	0.1 ± 0.03 a
9-cis-zeaxanthin	0.01 ± 0 b	0.02 ± 0 ab	0.02 ± 0 a
13- or 15-cis-β-cryptoxanthin	0.08 ± 0 c	0.23 ± 0.03 b	0.62 ± 0.01 a
α-cryptoxanthin	0.07 ± 0.02 c	0.15 ± 0.03 b	0.37 ± 0.01 a
β-cryptoxanthin	1.46 ± 0.21 c	3.02 ± 0.14 b	5.73 ± 0.3 a
β-carotene	0.10 ± 0.01 c	0.15 ± 0.03 b	0.41 ± 0.03 a
9-cis-violaxanthin-C12:0-C12:0	0.02 ± 0 c	0.03 ± 0 b	0.05 ± 0.02 a
9-cis-violaxanthin-C12:0-C14:0	0.04 ± 0c	0.08 ± 0.01 b	0.14 ± 0.05 a
9-cis-violaxanthin-C12:0-C18:1	0.17 ± 0.01 b	0.2 ± 0.03 b	0.99 ± 0.08 a
9-cis-violaxanthin-C14:0-C14:0	0.06 ± 0 b	0.08 ± 0 b	0.18 ± 0.04 a
Mixture of 9-cis-violaxanthin-C12:0-C16:0 and 9-cis-violaxanthin-C14:0-C18:1	0.15 ± 0.02 b	0.25 ± 0.04 b	0.94 ± 0.11 a
Mixture of Violaxanthin-C14:0-C16:0 and Violaxanthin-C16:0-C18:1	0.09 ± 0.01 c	0.15 ± 0.02 b	0.19 ± 0.02 a
β-cryptoxanthin-C18:0	0.06 ± 0.01 b	0.07 ± 0 b	0.39 ± 0.07 a
Total carotenoid	2.49 ± 0.07 c	4.61 ± 0.13 b	10.27 ± 0.74 a

Data are mean ± SD. Different lowercase letters represent significant differences, *p* < 0.05.

## Data Availability

No new data were created or analyzed in this study. Data sharing is not applicable to this article.

## Abbreviations

GAE, gallic acid equivalents; SSF, simulated saliva fluid; SGF, simulated gastric fluid; SIF, simulated intestinal fluid; CBA, carotenoid bioaccessibility; FC, fresh citrus; FCJ, fresh citrus juice; NCJ, not-from-concentrate citrus juice; HPH, high-pressure homogenization; TCC, total carotenoid content; CRR, carotenoid retention ratio; VC, vitamin C; SSA, specific surface areas; ASA average surface areas.
